# Vertebral osteomyelitis and epidural abscess due to *Listeria monocytogenes* – case report and review of literature

**DOI:** 10.5194/jbji-7-75-2022

**Published:** 2022-04-13

**Authors:** Olayinka Ibironke Adebolu, Jennifer Sommer, Abiodun Benjamin Idowu, Nicole Lao, Talha Riaz

**Affiliations:** 1 Department of Internal Medicine, Cleveland Clinic Akron General Medical Center, Akron, Ohio, USA; 2 Department of Radiology, University Hospitals Cleveland Medical Center, Cleveland, Ohio, USA; 3 College of Medicine, University of Lagos, Lagos, Nigeria; 4 Division of Infectious Diseases, University of Arizona, Tucson, Arizona, USA

## Abstract

We describe a case of native vertebral osteomyelitis (NVO) secondary to *Listeria monocytogenes* in a patient with polymyalgia rheumatica receiving chronic steroids. Treatment required surgical debridement of the epidural phlegmon and combination therapy with intravenous ampicillin and gentamicin.

## 1 Introduction

*Listeria* typically causes self-limited gastroenteritis; however, severe infections such as endovascular listeriosis and neurolisteriosis (brain abscess, rhombencephalitis) occur in high-risk patient subgroups such as pregnant women, immunocompromised patients, and those at extreme ages.

*Listeria* does not typically invade native joints; native vertebrae infection is even more rare. Bone and joint *Listeria* infections are primarily reported in those with prosthetic devices. We report a case of *Listeria monocytogenes* native vertebral osteomyelitis (NVO) and reviewed the published literature.

**Table 1:** Characteristics of cases of listerial NVO.

Author	Age Sex	Past medical history	Presenting complaint	Disease duration	Lab	Culture reported	Imaging	Treatment	Outcome
Chirgwin et al. (1989)	57 M	Diabetes mellitus, asthma, 50 mg prednisone for 4 years	Fever, back pain	3 weeks	WBC: $12.9\times 10^{9}$ L ${}^{-1}$	Bone fragment culture: positive	Myelogram showed anterior cord compression at T4–T5	Cord decompression, ampicillin, and tobramycin for 6 weeks	No evidence of infection at 6-month follow-up
Al Ohaly et al. (2020)	79 M	Bilateral subclavian to carotid artery bypass, infrarenal aortic aneurysm repair	Back pain	3 weeks	CRP: 87.9 mg L ${}^{-1}$	Bone aspiration and biopsy: positive Blood culture: negative	MRI: high signal intensity within L3–L4 disc space involving inferior endplate of L3, superior end plate of L4 with end plate irregularity	Ampicillin for 6 weeks	Rapid clinical improvement. No recurrence of disease at 2- and 6-month follow-up
Aubin et al. (2016)	92 M	Diabetes, heart failure, hip arthroplasty, gastric ulcer, smoking, alcohol use	Fever	1 week	CRP: 70 mg L ${}^{-1}$ ESR: 46 mm h ${}^{-1}$	Vertebral biopsy: unclear Blood culture: not reported Perianal abscess: positive	MRI: multifocal spondylodiscitis with global (L4–L5) and focal (L3–L4 and L5–S1) hypersensitivity of the discus	Gentamicin for 4 d and IV amoxicillin daily for 6 d, then co-trimoxazole for 3 months	At 3 months, mobility regained but slow recovery
Khan et al. (2001)	69 M	Prior spinal laminectomy	Back pain	5 months	WBC: $11.9\times 10^{9}$ L ${}^{-1}$ ESR: 58 mm h ${}^{-1}$	Epidural abscess: positive Blood culture: negative	MRI: synovial cyst at L3–L4 and an area of signal abnormality with mild peripheral enhancement at the L5–S1 level suggestive of epidural abscess	Surgical resection of abscess and laminectomy at L5–S1, followed by ampicillin and gentamicin	Not reported
Camp and Luft (1973)	67 M	Diabetes mellitus, multiple lumbar disc surgeries	Back pain	Not reported		Vertebral biopsy: not reported Blood culture: negative	Not reported	Oxacillin and streptomycin	Death
Hasan et al. (2017)	63 M	Diabetes mellitus, bioprosthetic aortic valve replacement	Back pain, Fever	2 d	CRP: 21 mg L ${}^{-1}$ WBC: 10 000 cells $µL^{-1}$	Blood culture: positive Resected valve culture: negative	MRI of lumbar spine suggestive of discitis and osteomyelitis at L4/L5 level, with associated right paravertebral muscle enhancement but no epidural abscess	Benzyl penicillin 14.4 g daily 6 weeks, then rifampicin 300 mg twice daily 4 weeks, then amoxicillin 1 g thrice daily 18 weeks	Complete recovery
Duarte et al. (2019)	65 M	Diabetes mellitus, alcohol use, smoking, dyslipidemia	Fever, lower limb weakness	5 d	CRP: 70 mg L ${}^{-1}$ ESR: 46 mm h ${}^{-1}$	Vertebral body biopsy: negative Anosacral abscess culture: positive	Lumbosacral MRI showed enhancement of fifth Lumbar vertebrae and sacrum	IV ampicillin 2 g 4-hourly for 2 weeks, the oral amoxicillin 1 g 6 hourly for 3 months	Clinical and radiological improvement from 2 weeks
Index case	60 M	Polymyalgia rheumatica treated with 20 mg of prednisone daily for the past year	Back pain	12 months	WBC: 14 200 mm ${}^{-3}$ CRP: 3.24 mg dL ${}^{-1}$ ESR: 64 mm h ${}^{-1}$	Blood culture: positive Epidural phlegmon: positive	Sagittal STIR MRI of lumbar spine showed an abnormal signal within the disc space and subtle signal abnormality along the endplates at L3–L4 as well as an epidural collection posteriorly at L2–L3	Ampicillin 2 g 4-hourly for 6 weeks and gentamicin 160 mg every 12 h for 2 weeks	Back pain had improved, and patient was doing well at 6 weeks

## 2 Methods

Embase, Google Scholar, and PubMed databases were comprehensively searched for the following terms: “listeria”, “listeria monocytogenes”, “arthritis”, “osteomyelitis”, “vertebrae osteomyelitis”, “spondylodiscitis”, and “discitis”. Reference lists of relevant articles were reviewed for potential studies. A total of nine cases have been documented: only seven reported in English language were examined in this study (Table 1). We excluded reports by Fernández de Orueta et al. (2011) and Schinagl et al. (2003) because they were in other languages.

## 3 Case report

A 60-year-old male grocery store worker from the Midwestern United States presented to the emergency room with acute exacerbation of lower back pain. He reported inability to get up on his feet and suffered multiple falls in the preceding months. The pain radiated down his right leg with associated right-thigh paresthesia. He denied fever but reported intermittent night sweats and new-onset loss of bowel control. His past medical history was significant for polymyalgia rheumatica treated with 20 mg of prednisone daily for the past year. A period of 6 months ago, he underwent endoscopy and colonoscopy in the context of chronic diarrhea. The stool study for enteric pathogens was negative. He had no personal history of diabetes, intravenous drug use, chronic cough, or weight loss. There was no history of travel outside the United States, and his HIV screen was negative.

On presentation, he was afebrile with a pulse rate of 101 beats min ${}^{-1}$ , blood pressure of 114/78 mmHg, and respiratory rate of 16 breaths min ${}^{-1}$ . The lumbar spine was tender. Muscle strength was 3/5 in bilateral lower extremities with normal sensation and intact anal sphincteric tone. His white blood cell (WBC) count was 14 200 mm ${}^{-3}$ , hemoglobin was 11.8 g dL ${}^{-1}$ , C-reactive protein (CRP) was 3.24 mg dL ${}^{-1}$ (reference: <1.0 mg dL ${}^{-1}$ ), the erythrocyte sedimentation rate (ESR) was 64 mm h ${}^{-1}$ (reference: 0–20 mm h ${}^{-1}$ ), creatinine was 3.24 mg dL ${}^{-1}$ , and the glomerular filtration rate (GFR) was 53 mL min ${}^{-1}$ 1.73 m ${}^{-2}$ . Two sets of blood cultures grew *Listeria monocytogenes* identified via matrix-assisted laser desorption/ionization time-of-flight mass spectrometry (MALD-TOF). The organism was susceptible to ampicillin (MIC 0.5) and penicillin (MIC 0.25). A transesophageal echocardiogram revealed no valvular abnormality.

**Figure 1:**
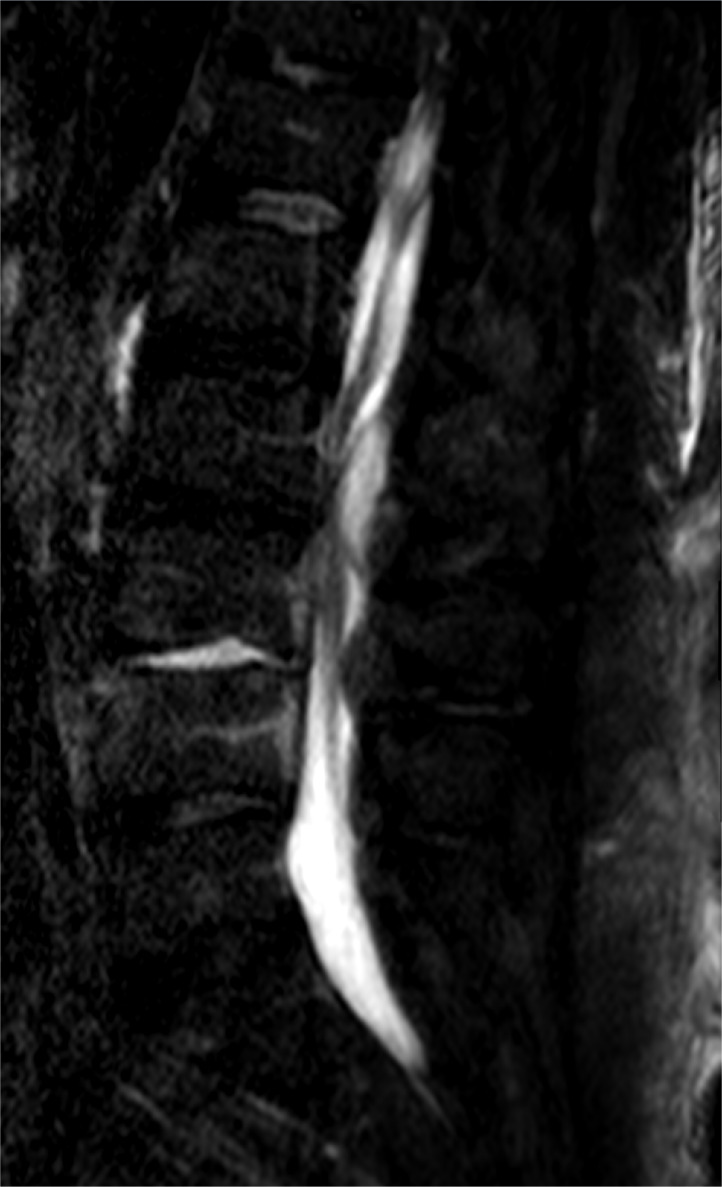
Sagittal STIR MR image of the lumbar spine showing an abnormal signal within the disc space and subtle signal abnormality along the endplates at L3–L4 as well as an epidural collection posteriorly at L2–L3 (originator: Jennifer Sommer).

**Figure 2:**
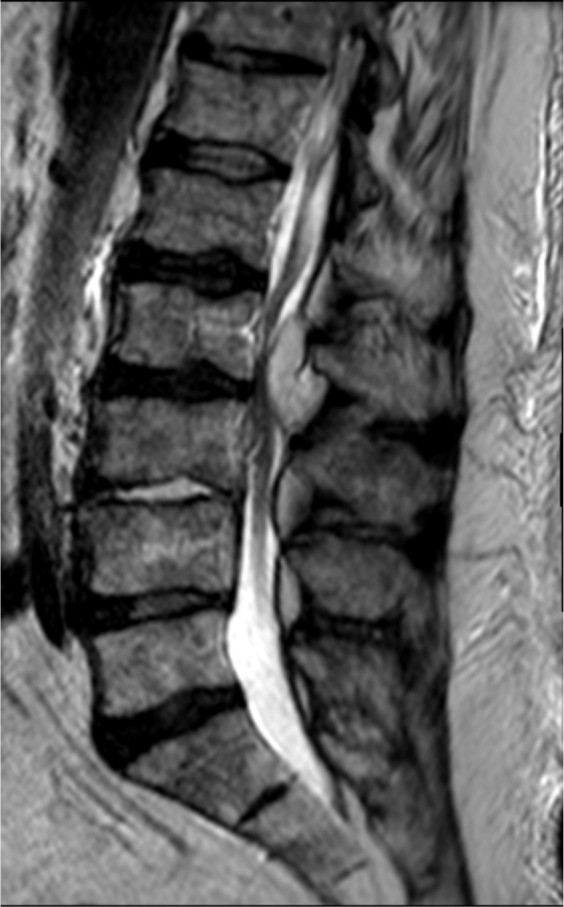
Sagittal T2 MR image of the lumbar spine again demonstrating an abnormal signal in the L3–L4 disc space and an epidural collection posteriorly at L2–L3 (originator: Jennifer Sommer).

**Figure 3:**
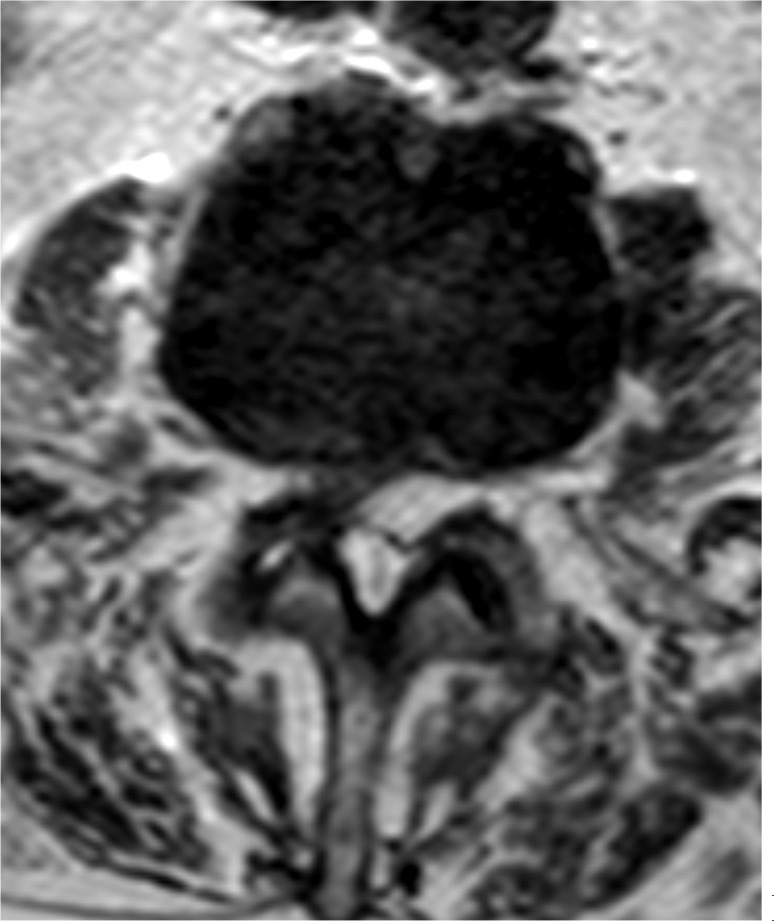
Axial T2 MR image at the level of L2–L3 showing an epidural collection centred posteriorly and to the left with mass effect on the thecal sac (originator: Jennifer Sommer).

A lumbosacral spine X-ray showed moderate multi-level lumbar degenerative changes. Non-contrasted Magnetic Resonance Imaging (MRI) of the lumbar spine revealed an abnormal signal within the disc space and subtle signal abnormality along the endplates at L3–L4 (Figs. 1 and 2). Additionally, there was a fluid collection within the epidural space posteriorly and to the left of the midline at L2–L3, with a marked mass effect on the thecal sac and severe canal stenosis (Fig. 3). These findings were concerning for discitis-osteomyelitis at L3–L4, with an epidural abscess posteriorly at L2–L3.

He underwent irrigation and debridement, including levels L2, L3, and L4 laminectomy and decompression of the phlegmon. Medial facetectomy and foraminotomy of L2–L4 and lateral decompression of the L2, L3, and L4 nerve roots were also done. Epidural phlegmon drained intra-operatively grew *Listeria monocytogenes*. A histopathologic specimen collected intraoperatively showed fibro-fatty tissue with acute-on-chronic inflammation and areas of tissue necrosis consistent with abscess tissue.

He was gradually tapered down from prednisone and discontinued. A peripherally inserted central catheter (PICC) was placed for the administration of ampicillin (2 g every 4 h for 6 weeks) and gentamicin (160 mg every 12 h for 2 weeks) at a skilled nursing facility. At 6 weeks post-treatment, the back pain had improved, and the patient was rehabilitating well. However, contrasted MRI of the lumbar spine showed post-surgical changes with evidence of discitis and osteomyelitis at L3–L4. At 6 months, subsequent non-contrasted lumbar spine MRI noted significant improvement in the previously reported L3–L4 disc signal and T2 hyperintensity.

## 4 Discussion

Listerial NVO is a rare manifestation because *Listeria* does not commonly affect the musculoskeletal system, except if the patient is immunocompromised or has in situ prosthetic material (Corrah et al., 2010). Established risk factors for *L. monocytogenes* include pregnancy, neonates and age above 65 years, hematologic malignancy, end-stage renal disease, recipients of organ transplants, HIV, alcoholism, and patients with auto-immune diseases on immunosuppression with steroids and TNF inhibitors (Del Pozo et al., 2013; Schett et al., 2005; Kubota et al., 2021). We hypothesized that use of chronic steroids and exposure to contaminated processed food predisposed our patient to this unusual infection. However, listeriosis can occur without identifiable risk factors (Al Ohaly et al., 2020; Aubin et al., 2016).

Our case suggests that listerial NVO has an insidious course of infection. The index patient had back pain for over a year; Khan et al. (2001) diagnosed their patient 5 months after the onset of intermittent backache. In a review of 43 cases of listerial bone and joint infections (BJIs), 73 % of infections were subacute or chronic in onset (Charlier et al., 2012). Additionally, in most documented cases (Table 1), compared to osteomyelitis secondary to *S. aureus*, inflammatory markers in *L. monocytogenes* NVO may be mildly elevated.

Blood cultures should always be obtained as many patients are placed on empiric anti-staphylococcal antibiotics, with a disc aspirate not always done (or it may be negative given receipt of prior antibiotics). The preferred imaging is an MRI of the vertebral column because of its high sensitivity ( ≥90 %) (Love et al., 2000). In four out of the seven reviewed cases (Table 1) and this index case (Figs. 1–3), contrasted MRI showed increased T2-weighted signal intensity of the affected disc space. An echocardiogram is warranted if there is concern for concomitant endovascular listeriosis as listerial endocarditis caries high mortality (Shoai-Tehrani et al., 2019).

Management of listerial NVO is based on clinical presentation. Surgical intervention may be warranted if there is a significant neurological deficit, cord compression, recurrence despite appropriate antimicrobial therapy, destruction of the vertebrae with instability, or large epidural abscess (Berbari et al., 2015). In patients where vertebrae is infected secondary to hematogenous seeding from an infected cardiac valve, antibiotics alone may suffice for the native valve, while replacement should be considered for an infected prosthetic valve (Kumaraswamy et al., 2018). Treatment includes intravenous ampicillin or penicillin combined with gentamicin to achieve a synergistic bactericidal effect. In those allergic to penicillin, trimethoprim/sulfamethoxazole is an alternative with comparable therapeutic efficacy (Temple and Nahata, 2000). Similar to listerial endocarditis and listerial brain abscess, the treatment duration for most *Listeria* BJIs is 4 to 6 weeks, though treatment duration to achieve a cure could be protracted in patients with delayed therapeutic response (Charlier et al., 2012). De-augmentation of immunosuppression is warranted in patients on immunosuppressive medication.

## 5 Conclusion

*Listeria monocytogenes* is a rare cause of NVO and typically has an insidious disease course. Receipt of chronic steroids or anti-TNF blockers is an important risk factor. Infection as a cause of back pain in an immunocompromised host must always be excluded.

## Data Availability

No data sets were used in this article.

## Appendix A Abbreviations

**Table:**

WBC	White blood cell
CRP	C-reactive protein
GFR	Glomerular filtration rate
ESR	Erythrocyte sedimentation rate
MIC	Minimum inhibitory concentration
